# Understanding Patients’ Decisions to Obtain Unplanned, High-Resource Health Care After Colorectal Surgery

**DOI:** 10.1177/10497323211002479

**Published:** 2021-04-10

**Authors:** Stephanie T. Lumpkin, Eileen Harvey, Paul Mihas, Timothy Carey, Alessandro Fichera, Karyn Stitzenberg

**Affiliations:** 1 The University of North Carolina at Chapel Hill, Chapel Hill, North Carolina, USA; 2 Baylor University Medical Center, Dallas, Texas, USA

**Keywords:** qualitative, emergency care, crisis, crisis management, health behavior, behavior, caregivers, caretaking, decision-making, determinants of health, information seeking, health, health seeking, users’ experiences, health care, Southeastern United States

## Abstract

Readmissions and emergency department (ED) visits after colorectal surgery (CRS) are common, burdensome, and costly. Effective strategies to reduce these unplanned postdischarge health care visits require a nuanced understanding of how and why patients make the decision to seek care. We used a purposefully stratified sample of 18 interview participants from a prospective cohort of adult CRS patients. Thirteen (72%) participants had an unplanned postdischarge health care visit. Participant decision-making was classified by methodology (algorithmic, guided, or impulsive), preexisting rationale, and emotional response to perceived health care needs. Participants voiced clear mental algorithms about when to visit an ED. In addition, participants identified facilitators and barriers to optimal health care use. They also identified tangible targets for health care utilization reduction efforts, such as improved care coordination with streamlined discharge instructions and improved communication with the surgical team. Efforts should be directed at improving postdischarge communication and care coordination to reduce CRS patients’ high-resource health care utilization.

## Introduction

Unplanned postdischarge health care utilization is common, costly, burdensome, and associated with lower patient satisfaction scores and inferior outcomes (Bliss et al., 2015; Damle et al., 2014; Leppin et al., 2014). According to a 2016 systematic review, rates of 30-day readmission after colorectal surgery (CRS) range from 9% to 25% (Damle & Alavi, 2016). Meanwhile, rates and impact of other postdischarge health care utilization, such as emergency department (ED) visits and observation stays, are largely unknown. As colorectal resections are the ninth most common type of surgery in the United States with more than 300,000 inpatient procedures performed annually, postdischarge health care utilization after CRS leads to substantial health care expenditures (“Most Frequent Operating Room Procedures Performed in U.S. Hospitals, 2003-2012 #186,” n.d.; Steiner et al., 2006). Readmissions alone after CRS result in US$300 million in expenditures annually (Wick et al., 2011). Beyond cost, readmissions after CRS are associated with high rates of reoperation, invasive procedures, intensive care unit admissions, delays of adjuvant treatment, and death (Damle et al., 2014). Consequently, there is significant national policy interest in decreasing the burden of readmissions, though current policy and the bulk of readmission research does not address this surgical population directly.

**Figure 1.**
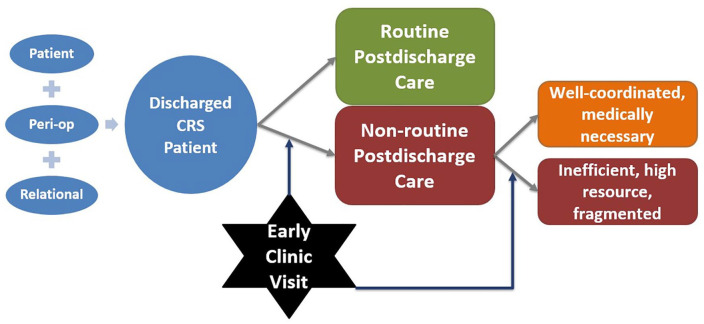
Conceptual model of the postdischarge health care utilization of a discharge CRS patient. *Note.* CRS = colorectal surgery.

There are several ways a patient can interact with the health care system after discharge, including (a) routine outpatient monitoring, such as clinic follow-up visits and telemonitoring; (b) ED visits; and (c) hospitalizations, either through readmission or through an observation stay (e.g., outpatient hospitalization lasting less than two midnights; Sheehy et al., 2013). Prior studies have shown that patients often feel vulnerable during the bridge period between hospital discharge and the first follow-up appointment (Kelly et al., 2016; Kripalani et al., 2014). While some postdischarge health care utilization events are medically necessary (e.g., for sepsis), many are likely avoidable (Lumpkin et al., 2018; Menendez & Ring, 2016). Postdischarge care can be well coordinated between the surgeon and the patient or can be fragmented and resource intensive. All these factors related to readmission have been well studied (González et al., 2017), though the decision to be readmitted is primarily a clinical decision. To be better conceptualize this, we developed a conceptual model—see Figure 1 (Andersen, 1995). Briefly, our conceptual model is based on the Modified Andersen Behavioral Model of Healthcare Utilization, where certain known factors play into how a patient makes a decision regarding their health care utilization after discharge. These include personal characteristics (socioeconomic status, basic demographics, and driving distance), as well as surgical or perioperative characteristics (type of surgery, surgical approach, and surgical complications), and finally relational or contextual characteristics, (relationship with the hospital or surgeon and a patient’s capacity to access their health care team). These personal characteristics are impossible to assess through database studies alone, and even survey-based studies cannot provide the richness and depth of information that interviews can provide. Nonetheless, given these characteristics, a patient can either have routine postdischarge health care utilization (no hospitalizations, ED visits, or complications) or they can have nonroutine postdischarge care (complications, hospitalizations, and/or ED visits). These nonroutine courses can be either medically necessary and well coordinated or they can be unnecessary, resource intensive, and/or fragmented.

It remains unclear how and why *patients choose* to seek out certain types of care, such as advice or in-person consultation with their surgical team versus seeking care directly in the ED. The purpose of this study was to characterize how and why patients utilize postdischarge care after CRS.

## Method

### Study Design

The study site is a large, tertiary care academic hospital with a catchment area of 100 urban and rural counties spread over three states, serving 37,000 patients each year. This study utilized participants from a prospective cohort of 150 adult patients undergoing colon or rectal resection between November 2017 and November 2018. For context, during this study window, all elective CRS patients at this institution participated in an Enhanced Recovery After Surgery (ERAS) protocol, unless they had a specific contraindication. The ERAS protocol and other local initiatives include elements of preoperative patient education, including what to expect after surgery. All adults older than 18 years undergoing CRS based on Common Procedural Terminology codes were included in this study, regardless of their surgical approach (minimally invasive or open) or their surgical indication (trauma, inflammatory bowel disease, cancer, infection, etc.). Patients were excluded from this study if they died during their index hospitalization or prior to their phone interview, if they had less than a 24-hour index hospital stay, or if they were discharged to a nonhome location.

Included participants were approached for written informed consent during their index surgical hospitalization, prior to discharge. Patients were not approached during periods of patient care, rest, or nonroutine floor status. For instance, participants were not approached for enrollment in this study while they were in the intensive care unit but were included after their care was advanced to routine-floor status. Baseline characteristics and covariates were measured at the time of enrollment, prior to discharge. The electronic health record was reviewed for postdischarge health care utilization. If, through chart review or through patient recollection during phone interview, they were identified as having been rehospitalized or seen in an ED without a readmission within the 30 days after discharge, this was recorded. Combined, these encounters formed our primary composite outcome, 30-day unplanned postdischarge health care utilization. Using this information and the baseline demographics, we used purposeful stratified sampling to complete our semi-structured in-depth interviews.

### Participant Selection

**Figure 2.**
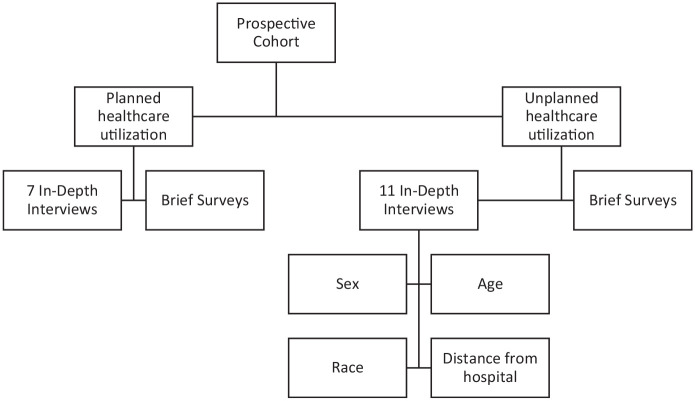
Purposeful stratified sampling technique to ensure richness and diversity of participant responses (planned). *Note.* Purposeful stratified sampling technique to ensure richness and diversity of colorectal surgery patient responses regarding their utilization of postdischarge care. Consecutive colorectal surgery patients were approached and enrolled in this qualitative study while in the hospital. They were then classified as having planned or unplanned health care utilization in the 30 days following discharge, as we wanted both experiences represented. We then purposefully sampled for variations in demographics such as age, sex, race, and distance from the hospital to improve diversity.

We attempted to call every eligible and consented participant in our cohort (*n* = 150) between 30 and 40 days after discharge. We were able to reach 98 (65.3% response rate) enrolled patients by phone with a median time frame of 81 days (interquartile range [IQR]: 66–107 days) with a mean 1.52 phone call attempts made (*SD*: 0.79). We attempted to contact patients at least 3 times at different hours of the day and days of the week at their preferred telephone number. Once contact was made, the participants were given the opportunity to reschedule or alter their level of participation. Three participants withdrew consent to participate after enrollment (one enrollee felt the questions were too invasive, two enrollees felt too tired to participate). A brief screening survey was used to determine whether to complete a long interview based on the purposeful sampling criteria (Figure 2).

Ultimately, 18 participants were selected for a single in-depth semi-structured interview based on purposeful stratified sampling with the goal of maximum variation of responses (Guest et al., 2006). Purposeful sampling allows researchers to “select information-rich cases related to the phenomenon of interest,” (Guest et al., 2006; Palinkas et al., 2015, 2012; Sandelowski, 2010; Sandelowski & Leeman). Specifically, we oversampled patients who were readmitted, visited an ED, or responded to our screening survey with eager or lush responses. This sampling technique was used to capture the diverse complexity of postdischarge courses and outcomes, but we did want to ignore valuable insight that patients with routine postoperative courses might provide. We conducted interviews from a secure workplace location and they were instructed to find a quiet and comfortable place for an in-depth phone conversation. Participants were allowed to have other caregivers, friends, or family members present at the time of the phone interview.

### Instruments and Data Collection

All participants underwent a 3-minute screening survey to assist with classification of the primary outcome. The survey consisted of three questions regarding the patient’s health care utilization since surgery, including visits to locations other than the index surgical hospital, and whether they perceived their postdischarge course to be straightforward, have a few bumps in the road, to be complex, or to be a nightmare. These categories were utilized to generate discussion in a colloquial manner that might highlight patients who have an interesting story to tell, based on their own perceptions, despite screening “negative” for our outcome of interest. We included patients from all categories in our study. In addition, the interview guide contained a brief series of validated survey questions to assess health literacy (Sarkar et al., 2011) and to screen for depressive symptomatology (Pfizer, 1999) both of which have been shown to influence how patients access care.

An interview guide was developed using deductive codes and concepts generated through literature review and expert opinion (Supplemental Content—Interview Guide). The topics were generated based on a conceptual model (Figure 1, see introduction for extended description) which was adapted from an existing theoretical framework as part of a larger mixed methods study. The interview guide, based on the conceptual model, was pilot tested and, through an iterative process, was improved for clarity throughout the first five interviews.

All in-depth interviews were audio-recorded with participant consent. Field notes and memos were made during and immediately after each interview to capture contextual observations. Each interview lasted approximately 45 minutes, although an hour was allotted. At the conclusion of the interview, participants were not recontacted, and they did not have the opportunity to check the transcripts or results for clarity. We did not recontact participants primarily because of the original consent and the Institutional Review Board (IRB) obtained did not explicitly request additional follow-up. There were minimal issues with the transcripts themselves, and where necessary, the audiofiles were reviewed by the research team. Participants were not blinded to personal goals of the researchers or reasons for conducting the study. Interviews were triangulated against electronic health records to ensure validity of dates. Follow-up and probing questions were generated to elicit completeness of response. Theoretical saturation for the primary research objective was a goal of the study and was assessed and obtained through systematic data analyses. Specifically, using rapid analysis, the coding team conducted preliminary analyses after 9, 12, 15, and 18 interviews to refine any interview questions and prompts and to assess whether any new information was obtained with addition of further interviews (Denzin et al., 2006; Guest et al., 2006; Ness, 2015). Briefly, rapid analysis is a data condensation process that allows for the answers to each interview question to be compared across a summary template that is brief enough to facilitate team-based discussion, but thorough enough to understand the complexity of the interview (Hamilton, 2018; McMullen et al., 2011; E. Sobo, 2003; E. J. Sobo et al., 2002; Solomon et al., 2007). After rapid analysis of all 18 interviews, we obtained extensive richness of responses and ended the data collection based on theoretical saturation.

There were two interviewers in this study.^1^ Neither interviewer had any relationship with the participants outside the context of the study. Interview transcripts were reviewed frequently with a qualitative research expert to refine interview techniques to ensure elaborated responses and to reduce bias.

### Coding and Analysis

All interview recordings were transcribed by a third-party, Health Insurance Portability and Accountability Act (HIPPA) and university-approved vendor and coded by two coders. This interpretive qualitative study aimed to systematically organize responses into a structured format (M. Sandelowski, 2000, 2010) All transcriptions were initially read for familiarity. Preliminary coding of interviews included brief memos regarding the interviewer’s potential biases, initial reactions, or any contextual clues. These memos, in addition to in vivo coding (i.e., using participant language to name the code) and inductive coding were used to create and refine the codebook during the first cycle of coding. Participant quotes were identified as representative of various codes. Multiple first-level codes were organized into a coding tree to generate higher level concepts or themes in the second level of coding, which mirrored and expanded, or branched the domains of the conceptual model (Figure 2). Through an iterative process, we refined several concepts such as the patient decision-making style (Saldaña, 2009). Descriptions of each overarching and patient-centered theme, or patterns within these data, were documented in the codebook to ensure well-defined and reproducible coding.

Specifically, our analysis blends descriptive coding of emotional responses and rationale with interpretive coding of decision-making processes to generate themes, or concepts. Primary coding of all interviews was done by dual-coding from each coder, who identified recurring codes and overarching themes. Next, a coding-consensus meeting was conducted to allow for refinement of codes and resolution of any disagreements. The application of codes was reviewed for discrepancies, which led to significant insight into the results based on researcher triangulation—using researchers from different multidisciplinary backgrounds on the analysis team. For example, coder A analyzed content from a surgical background, which was well complemented and balanced by the public health background of coder B (Landau & Drori, 2008)

These data are not publicly available due to their containing information that could compromise the privacy of research participants. All coding and qualitative analyses were completed using QSR NVivo 12 Software.

## Ethics

This study protocol was reviewed and approved by the IRB of the University of North Carolina, and all participants provided informed, written consent prior to study participation.

## Results

Overall, we conducted 18 in-depth interviews (see Table 1 for participant characteristics). The interview participants ranged in age from 21 to 78 years. Seventy-eight percent were White, non-Hispanic, and 50% were female. The primary indication for surgery was diverse (40% cancer, 40% inflammatory bowel disease, 20% other). Two participants screened positive for low health literacy. When asked about their perception of the postoperative period, two participants described their time since discharge as “straightforward,” seven as “a few bumps in the road,” seven as “complex,” and two as a “nightmare.” More than half of the participants (56%) lived further than a 1-hour drive from the hospital. Fifteen (83%) had attended a clinic visit with their surgeon prior to the interview. We purposefully oversampled patients with complex postdischarge health care courses. As a result, 11 (61%) participants had an unplanned high-resource health care utilization event within 30 days of discharge, with the majority of these (55%) being readmissions.

**Table 1.** Participant Demographic and Medical Characteristics.

Characteristic	Entire Prospective Cohort (*N* = 150)	Interview Participants (*N* = 18)
Sex, *N* (%)		
Female	80 (53.3)	9 (50)
*M* age in years (range)	43 (20–82)	56 (21–78)
Ethnicity, *N* (%)		
White, Hispanic	3 (2)	1 (5.5)
Black	29 (19)	3 (16.5)
White, non-Hispanic	110 (73)	14 (78)
Other	8 (5)	0 (0)
Presence of an ostomy, *N* (%)		
Yes	29 (19)	8 (44)
Attended a follow-up clinic visit with their surgeon, *N* (%)		
Yes	130 (87)	15 (83)
30-day unplanned postdischarge health care use, *N* (%)		
None	102 (68)	7 (39)
Emergency department visit	9 (6)	4 (22)
Observation stay	5 (3)	1 (6)
Readmission	32 (21)	6 (33)
Low health literacy, *N* (% of respondents)		
Yes	29 (30)	2 (11)
Driving distance from surgical hospital, *N* (% of respondents)		
Less than or equal to 1 hour	72 (73)	8 (44)
Description of postoperative period, *N* (% of respondents)		
Straightforward	45 (48)	7 (39)
A few bumps in the road	31 (33)	7 (39)
Complex	14 (14)	2 (11)
Nightmare	4 (4)	2 (11)

### Patient Decision-Making

After ensuring a diverse group of responses through purposeful sampling, we found that patients used several strategies for decision-making that they used or would have used when deciding whether and how to seek unplanned care. Some patients used detailed, process-level language, for example, if/then statements with a clear order of steps to handle each postoperative problem or scenario:

I think probably the first thing is I would identify my symptoms. If I just was—were having chills, I would take my temperature, see if I have a fever. Things like that. If I did have a fever, then I probably would call the on-call doctor at night. If I didn’t have a fever, I would probably wait it out till the morning and just see if anything got better. If I had any increase of pain, if I was nauseous, or if I was throwing up, I would definitely go to the hospital regardless.

Other patients were unable to give detailed process steps for making a decision, but deferred the decision to a caregiver, discharge paperwork, or their surgical team for advice.

Well, if I was having a problem, I would call someone . . . and they would tell me what to do or do I need to go back or something like that.

Finally, some patients could not describe any process for assessing their symptoms and seeking out care.

I, uh-uh—if I feel like I want—I need to go to the emergency room, I ain’t gonna call first. I’m gonna—I’m gonna go and take off. Uh, every second counts.

Notably, all participants were able to be categorized, primarily based on their decision-making strategy, such as *algorithmic, guided, or impulsive*. It was at this stage that participants had the opportunity to assess their own medical needs. Algorithmic patients had higher levels of health literacy and more exposure to the health care system, and thus were able to more accurately assess the severity of a perceived complication. Guided patients were also able to assess severity, if it was a complication that had been discussed in their discharge education or paperwork. Impulsive patients describe making their decisions from a place of helplessness, franticness, and vulnerability, often leading to impulsive or rushed decision-making. We struggled with finding a term that encompassed this type of frantic decision-making which often stemmed from a disempowered state and led to little room for intervention. These participants did not describe a particular instinctiveness, ease, confidence, or comfort with their decision-making. While impulsive may seem pejorative, it captures the fact that participants did not describe a particular instinctiveness, ease, confidence, or comfort with their decision-making. We believe that this term stays close to the data and most accurately describes how patients describe their decision-making.

We classified each interviewee’s overall decision-making style as algorithmic (*n* = 5), guided (*n* = 9), or impulsive (*n* = 4) The baseline characteristics of patients in each group are shown in the Supplemental Data. Overall, compared with guided patients, impulsive patients were less likely to have nonroutine postdischarge care (2 of 9 vs. 3 of 4). When impulsive patients did have nonroutine postdischarge care, it was more likely to occur at a nonindex hospital (3 of 4) compared with guided patients (2 of 9).

### Specific Patient Rationale for Going to the ED

Participant responses were also coded for presence of specific rationale regarding decision to seek or avoid unplanned high-resource health care after CRS. Nearly all patients identified a specific justification or rationale. Justifications were based on prior personal or familial health care experiences and were used to either avoid or seek out nonroutine postdischarge care. Some patients sought out health care because they acknowledged medical needs that were specifically concerning,

Uh, if-if my rectum started bleedin’ or somethin’ like that . . . or somethin’ wrong with my body that no, I can’t fix it myself, I know I need to go to the emergency room.

In this case, the patient was particularly concerned about his bloody stools and felt that going to the ED was the most expeditious way to address this problem, whereas other patients (*n* = 3) were driven by a fear of progression of symptoms:

You better go ahead and get that checked out before it turns into something serious.

In these three cases (two algorithmic and one guided patient), the threat of progression of symptoms led patients to visit the ED, despite feeling relatively stable currently.

Pain, in particular, was difficult for patients to accurately assess. Specifically, when patients worried about pain, they felt compelled to visit the ED:

When I first got out, I really struggled with intense pain. I was convinced something was wrong. I was having to go to the [ED] for it. They couldn’t figure it out. I really thought that I had an obstruction. . . Obviously, as a patient, you panic because you don’t want anything to be wrong. Having to keep going back and forth, then not being able to find anything, was the hardest . . .

When patients did arrive to the ED for pain control, they were often disappointed with the pain control that they received, how they were treated, or the threat of being labeled a drug seeker:

I think pain’s always very tricky when it comes to the hospital setting. I know that just from being a chronic—having a chronic illness and going to the ER for pain. Not because of surgery, but just for different things. Also, being 21-years-old, I’m in college. You can get labeled as drug-seeking, when that’s not it at all. It’s, first of all, very, very frustrating.

Specifically, some patients felt that their pain was ignored, downplayed, or labeled as drug seeking. In addition, even when their pain was addressed satisfactorily, they found the entire experience in the ED to be chaotic:

Well, I just don’t see a need to do that [go to the ED]. It seems like there’s always a lot of chaos there, and a lot of extended waiting. I’d rather go directly into admissions if I’m going to have to be there.

Overall, more algorithmic and guided patients agreed their surgeon and surgical hospital was the best place to seek care:

Well, honestly, where we live in . . . that area down the coast. [That non-index] hospital has some great oncologists and great surgeons. They just were not able—they didn’t have the equipment and were not able to handle the kind of surgery.

Beyond the patient’s surgeon, several patients identified other providers, including primary care physicians and medical specialists that they could reach out to prior to visiting the ED:

He’s gonna probably tell them the same thing in the emergency room, sitting all that time waiting, spending a whole lot of money. You can go to your primary doctor, and they tell you the same thing.

They placed a premium on the medical expertise of their own primary care physicians and specialists and knew that these individuals could provide specialized and tailored medical advice, in a way that is impossible for the ED to provide. In addition, a few patients saw the ED as an undesirable alternative, even to the extreme, suggesting that they would only go to the ED for a life-or-death emergency:

I would rather be shot than go to the emergency room. I would rather wait it out unless I was having a heart attack, a real emergency. I’d rather wait until I can get better quality care with my own doctor.

Some patients made different decisions about how to proceed with their health care utilization, despite the same complication. For instance, when discussing a hypothetical or actual fever, participants with a prior negative experience in the ED stated that they would favor calling their surgeon first prior to presenting in the ED. Alternatively, a patient without a prior negative ED experience justified going to the ED immediately for a fever.

Two participants were unable to give any particular reasons or rationale for seeking out or avoiding the ED. In one example, the participant had minimal experience with the health care system prior to surgery, screened positive for low health literacy, and was categorized as having impulsive decision-making in regard to health care utilization.

### Emotional Responses to Care Needs

A participant’s decision to seek out or avoid unplanned high-resource health care was based on their emotional self-reflection into their ability to care for themselves after surgery. Several patients expressed frustration with their overall postdischarge situation or care coordination which led them to reconsider utilizing routine health care appropriately:

You definitely get frustrated and you just wanna be like, “You know what? I’m not gonna call them.” At the same time, while I would think that, I would still do it ‘cause I knew that was what was best for me. I think that’s the important part, is you have to remind yourself that’s what’s important.

Several patients discussed a sense of vulnerability, fear, or being overwhelmed which made them reconsider utilizing routine postdischarge care, including one older adult woman who previously felt quite confident about her health care decisions:

I feel more vulnerable now than I did before. I’d never had surgery before.

In addition, a previously healthy White male discussed his new sense of feeling overwhelmed:

I was able to get the visiting nurse to come out. She wasn’t scheduled till Monday, but I needed her on the weekend . . . [If she hadn’t been able to come, I would have] sat there and bawled my eyes out, probably.

Several patients confirmed this sentiment that surgery brings up a lot of new emotions making it difficult to make levelheaded decisions:

Emotionally. That was very unexpected after the very first surgery. That just hit me like a brick wall. At least after second surgery, I was like, okay, I’m gonna be a little emotional after this. My wife’s like, “Look, you’ve just had your guts ripped out. Your body, all the hormones are shifting around.”

Each participant also discussed a certain magnitude of emotion that either overwhelmed their decision-making (strong) or that they were able to “temper” with preexisting rationales or decision-making processes.

### Overview of Facilitators and Barriers to Optimal Postdischarge Care

Given the framework for decision-making that emerged from our in-depth interviews, we further asked participants about explicit barriers and facilitators to avoiding unplanned high-resource health care utilization. Specific facilitators to seeking out well-coordinated, medically necessary care include a strong support network, high health literacy, clear expectations, strong sense of self-efficacy, ease of communication with the surgical team, and belief that planned follow-up care is superior to unplanned care. When asked about barriers to well-coordinated care in the postdischarge period or specific reasons why they sought out unplanned, high-resource care, participants cited caregiver burden, limited finances, limited time, ongoing symptoms that preclude travel, travel distance, inability to contact the surgical team, and unclear expectations at discharge.

### Facilitators

Several patients (*n* = 10) gave enormous credit to their *caregivers and support network* for going through the recovery process with them and helping them make health care utilization decisions:

Well, it’s been a new experience for both of us [my wife and myself]. It’s involved a lot of effort on her part. She’s really been a big, big help, especially in the early stages when I couldn’t do too much for myself . . . I really appreciate everything’s she’s done during this time. I’ve tried to let her know that too.

In addition to these personal relationships, several patients (*n* = 5) discussed the *ongoing relationship with their surgeon and surgical team* as critical to the ability to access routine health care:

Anytime we ran into something out of the ordinary, we were able to reach—I don’t know what her title is, but the nurse practitioner for [our surgeon] and she was back with us immediately with answers.

For 16 patients, this relationship was built on mutual respect and trust, which allowed for *authentic communication*:

Dr. [Surgeon], [the nurse coordinator], his staff . . . they’ve become friends of ours . . . I respect all of them a great deal. I think we’ve earned their respect as well. We know they’re doing the best they can, and we have to do our part . . . The mutual respect is, I think, it begins with trust . . . I think they trusted us to do our part and to also be open and direct in our communications.

In addition to this personal relationship, one patient echoed the importance of *clear expectations and written communication* with this team. In particular, this patient discussed how this information can be used to evaluate and put his personal experience into context, which helped him to triage his own needs appropriately:

I think it’s definitely very helpful to know of the things that could happen. While, yes, it could be scary and overwhelmingly, it’s definitely better to know about them because if you didn’t, then it could be a lot worse. You might ignore it and think, “Well, I just had surgery, that’s probably why it’s happening.”

Another patient echoed this sentiment and specifically found the *reassurance and discharge teaching* that he received in the hospital as invaluable during the vulnerable postdischarge period:

Obviously coming out of surgery, it’s a very vulnerable time for a patient. It could be very hard. For this surgery in particular, you are losing an organ. It can be kinda scary because you don’t know about the next step, but the hospital staff were amazing about reassuring me that everything—they were going to be on everything.

While most interviewees (*n* = 17) discussed the importance of others in making their decisions, several discussed the importance of *autonomy, high health literacy, and personal responsibility*, suggesting that these are irreplaceable. One older adult woman discussed her own optimism and capacity when discussing things that made obtaining routine health care easier:

Probably the fact that I’m a very positive person. I’m attentive. I listen. Even though I’m 78, I still run my businesses, and have my mental faculties. It was a pretty obvious procedure. Nothing was to the point that it wasn’t understandable.

Notably, this patient did not personally acknowledge this privileged standing compared with many patients or specifically how this helped her navigate a complex health care system after surgery. Another patient states the importance of autonomy, but instead of the *pull yourself by your own bootstraps*, this patient discusses how all the tools were given to him at discharge, and then it was up to him to use them fully:

So when you get home . . . So, you know, ain’t no nurses gonna come and help you. So I think, um—I think they taught me a lot while I was in the hospital. So I think I’m okay now.

Finally, all the patients who only obtained routine postdischarge health care (*n* = 7) ultimately believed that this *routine care had inherent value in their postdischarge recovery*. When asked how he fits routine follow-up appointments into his busy schedule, one patient said the following:

[Routine follow-up appointments are] a priority. I make that sure I don’t miss them. I had not had to reschedule them. I’m going to work my personal life and my business life around them. There’s been no conflict to this point.

Another patient echoed this and specifically said that these appointments were a way to hold himself accountable, and he ultimately attributes this surveillance to his uncomplicated recovery:

I know that you need accountability in anything, and I think they’re holding me accountable for doing my part. They had these regular checks where we review and make adjustments if we need to. I think that if weren’t having these regular appointments at intervals then it would be easy to get off track.

### Barriers

Despite the fact that most patients (*n* = 16) would have chosen to go to their surgeon instead of seeking nonroutine health care, a few acknowledged (*n* = 3) in hindsight that several barriers would force them to pursue nonroutine health care over routine health care. Several *logistical issues* played into whether a patient could seek routine versus nonroutine care, such as worsening symptoms in the middle of the night:

I did [think about coming back to the hospital], but 11:00 at night and a three-hour drive, not feasible.

Even when some patients tried to contact their surgeon and avoid nonroutine care (*n* = 5), it was sometimes difficult to reach a provider during an inopportune time, such as the *middle of the night*:

If it was at night, and I would have to call and say, “Hi, I have a fever, this is what it is. Should I come into the ER or should I wait it out? What regimen should I do?” I think the hardest part is honestly getting in contact with the doctor. Especially at night because you won’t be talking to your specific doctor. If they, obviously, can’t pick up right away and the operator said that they’ll give you a call, there’ve been a few times that they never called me back and I ended up just having to go straight to the ER.

Sometimes, the decision to seek nonroutine care at local ED versus returning to the index surgical hospital was pragmatic. One patient simply felt physical burden, such as being *too sick to spend extra time in the car*:

The drawback to driving that far to [the hospital] would be what you go through on the way back . . . You’ve got the nausea, you’ve got the diarrhea, you’ve got all these things, and you’re spending two and a half hours on the interstate. It becomes bad.

In a world of limited time and resources, one patient lamented about the perceived *limited utility of a routine follow-up* visit:

Then I got this next [follow-up appointment], it was just on MyChart . . . it came up that I had another visit schedule with an NP, and I thought, the follow-up physician who saw me hardly did anything other than look at my scar. What was an NP gonna do for me? I decided to save United Health Care money, and I just canceled it.

Several patients (*n* = 4) worried that their *caregivers were taking on too much burden*, and weighed this guilt into their decisions to attend appointments or seek care:

There was a point our entire world revolved within 60 feet of my bedroom. My wife just got in the car. It was that way for how long? Six months or seven, wasn’t it? To where your whole life—and you get to the point where you feel like you’re a prisoner at home.

Another patient placed these routine and nonroutine health care decisions in the context of a very tumultuous and difficult phase in his life, where he was adapting to no longer being financially independent and feeling like a *personal burden to his family*:

The whole process is so complicated and takes so long to do. [The doctor] filled out the paperwork and [nurse coordinator] helped spearhead getting everything done. Once we got this going, then you got to the point where you’re [explicative] ‘cause it takes five or six months for the Social Security to kick in. You’ve got that five or six months that you’ve got to figure out some way to live. We managed for five months to live off of $750 a month SSI, food stamps, and my wife busting her tail at Food Lion grocery store as a cashier. Then as a man, you get to the point where you just—you’re burdened. That was the hard thing.

### Targets for Intervention

Finally, we asked participants about what things did or could have kept them from utilizing the ED or going to the hospital directly. Participants were asked the following: “What could be done to prevent you from going to the ED after surgery?” Some responses were spontaneous, whereas other participants required follow-up probes, such as, “If you had access to [a particular intervention] would that have been helpful?” The interventions generated were coded from participant interviews and organized into several distinct targets. The various interventions were discussed in both positive tones, such as expressing appreciation for well-done care, and more negative tones, such as concern or sense of lacking in a particular aspect of patient care. Patients were hungry for very tangible and transactional components of their discharge planning. There were several specific categories of interventional targets, including clear expectations, clear communication, ease of communication, ease of access to care, and reliable and ongoing support from both their provider and the support network. Specifically, for example, some patients stated that they did not know their postdischarge care was a priority, and they wanted very concise written instructions about how to care for various needs such as their ostomy, drains, or new medications. They described wanting phone and electronic access to a member of their surgical team at all times of day and being able to be seen quickly if a problem arose.

While the patients gave very tangible targets for intervention, we believe that these suggestions represent several higher level concepts. First, patients expressed a desire for empowerment through knowledge of their condition and a clear understanding of the road to recovery. In addition, the emphasis on clear and easy communication suggests that patients want to be considered mutually engaged active stakeholders in their discharge planning. They also felt that having easy access to appropriate care led them to truly having agency over their postdischarge health care needs, which was a source of motivation. In addition, these data suggest that patients are often willing to acquiesce to the advice of their surgeon in terms of how to triage their medical needs, especially after they have developed the ongoing and intimate relationship that is inherent to surgery. Nevertheless, having a clear understanding of postdischarge expectations allowed patients to take a large stakeholder share in the recovery process.

## Discussion

Our findings indicate that adult CRS patients rely on previous knowledge, through personal experience, the experience of others, or directed guidance to make decisions about how and when to access health care after surgery. Our findings are consistent with several conceptual models in the decision-making literature, namely that there is significant interplay among a patient’s personal factors (socioeconomic and demographic factors), interpersonal factors (relationships with support network and surgical team), capacity to make a certain decision (understanding of consequences), and the actual decision-making process. When these factors do not align, this can lead to significant decisional conflict, though the regret and confusion that underlies this type of conflict has only been studied in terms of treatment decisions, not in relation to seeking various types of health care (Thompson-Leduc et al., 2016). We believe that decisional conflict helps explain why some patients may seek “suboptimal” care (i.e., going to the ED independently and directly). They may believe that the “suboptimal” care, such as going to the ED without consulting the surgical team, is superior, medically necessary, or more convenient. Furthermore, patients who did not go to an ED verbalized the inverse of this; they were able to successfully navigate optimal care because they knew to only use the ED for emergencies and they had clear guidance about where to go after surgery. In addition, during this often life-altering surgery, some patients may experience what a recent qualitative study describes as *traumatic distress* related to the uncertainty of their illness (Coelho et al., 2020). This was especially true for patients with new diagnoses such as colorectal cancer or unexpected sudden onset of severe illness, such as diverticulitis. We found that these CRS patients also describe a type of traumatic distress which leads to anticipatory grief (Coelho et al., 2020), a concept that helps encapsulate many of the sentiments and uncertainties that patients felt about their transition back home.

We found that participants were generally amenable to considering different options for health care utilization. This is consistent with intervention theory literature, which posits that as many stakeholders as possible need to be in agreement about the choices available to change behavior (Fishbein et al., 2001). Although, in our study, we found that patients were more likely to acquiesce control to their surgical team. In a seminal article, Axelrod eloquently explains why surgeon–patient relationships are inherently different from other doctor–patient relationships:

The invasive and potentially life-threatening nature of surgical therapy fundamentally shapes the relationship between a surgeon and his patient and requires an extraordinary degree of trust from the patient and, correspondingly, ethical action by the surgeon. Through the evaluation and therapy of a patient’s condition, the power and control of the clinical encounter is gradually transferred from the patient to the surgeon . . . This transfer of power and control differs substantively from the power dynamics between patients and practitioners in most other fields of medicine. (Axelrod & Goold, 2000)

In our study, the patient, caregiver, surgeons, and additional providers need to understand, work toward, and communicate a shared goal of reducing suboptimal health care utilization (Fishbein et al., 2001). Therefore, patients and families may benefit from greater clarity on the indications for different types of postdischarge health care utilization.

While current transitions of care frameworks focus heavily on discharge education for patients with chronic medical needs, such as congestive heart failure, there is little consensus or clarity on what discharge education and preparation is needed for postsurgical patients. Our study reaffirms that a patient’s prior medical needs and their transition back home are key drivers of their individual health care utilization patterns (Andersen, 1995; González et al., 2017; Kelly et al., 2016). Specifically, our data suggest that this education, which is typically nurse driven, should include anticipatory guidance about how and when to use the ED. Several studies have examined how a patient’s perceived readiness for discharge can affect their readmission rate, and potentially even their ED visit rate, though these were not specific to surgical patients. In a 2017 qualitative study of CRS patients, patients stated that the discharge instructions, when complete and accurate, served as “a sense of security, a reminder of in-hospital education, a living document, and a source of empowerment” (Horstman et al., 2017). In our own departments’ quality improvement work and value stream mapping, nurses have stated that this document is a blueprint for discharge education (Lumpkin et al., 2019). Finally, in a nurse-driven randomized trial, the ReEngineered Discharge project found that a standardized discharge process, paperwork, and delivery of education materials was associated with a decrease in rehospitalizations and ED visits among adult nonsurgical patients on a teaching service (Jack et al., 2009). Our study confirms that the discharge paperwork and subsequent patient education is a key component of discharge planning. While the discharge paperwork can be considered a decision support tool, our study participants added that, in addition to being a high-quality standardized pile of paperwork, the discharge instructions—both verbal and written—would need to address several key relational components of decision-making—*attitudes* toward optimal and suboptimal care utilization, *normative pressure* to avoid suboptimal care and to seek out optimal care as a sense of duty and ownership in care, *self-efficacy* to manage and identify postsurgical issues, and *intentions to communicate* effectively with the surgical team (Frosch et al., 2009).

Our findings suggest that CRS patients are yearning for a partnership with their surgeon that can allow for a brokered decision to seek health care. In the inpatient setting, a 2017 *JAMA Surgery* article examined the role of shared decision-making between older adults facing complex surgical conditions and their surgeon as a means to improve value-concordant care (Taylor et al., 2017). In 2018, a survey-based study linked poor shared decision-making with an increased risk of ED utilization (Hughes et al., 2018). The surgical team in this role becomes an advocate and guide for the postdischarge health care utilization of the patient, in addition to their shepherding of the preoperative and inpatient process.

Ultimately, given these findings, future work should be aimed at implementing these tangible solutions and exploring the role for shared patient decision-making prior to unplanned high-resource health care utilization. In addition, validated methods for creating clear discharge instructions and effective decision-support tools for patients, especially those with low health literacy or no prior exposure to surgery, are needed.

There are several strengths to the study. First and foremost, the richness and diversity of responses represent the vast differences seen among various CRS patients. Second, the merging of clinical and qualitative expertise led to nuanced methodology and triangulation of disciplinary perspectives. Third, the study was designed using a conceptual model, grounded in the existing literature, to assess the multilevel factors associated with health care utilization after surgery. Finally, this research is relevant; although readmission rates have decreased after several intensive years of focus, patients still have ongoing health care utilization needs after surgery and the burden of unplanned high-resource health care utilization, such as ED use, after CRS remains substantial (Ibrahim et al., 2017).

One unanticipated concern with this study was the difficulty in contacting patients within the initial 30 to 40 days after discharge. Given our desire to improve retention of patients and reduce selection bias, we chose to continue attempting to contact participants. This may have led to a recall bias, as some individuals were interviewed as much as 3 months after their discharge. In addition, this study did not specifically examine presurgical health care utilization as a quantitative covariate. Despite this potential limitation, we did specifically target diverse patient groups (inflammatory bowel disease vs. cancer) to help elicit some of these subtleties. Another potential concern of this study is an element of social desirability bias, but due to our rapid analysis approach and having two trained interviewers, we believe that we obtained thorough and honest responses to our questions. This study may not be generalizable to some of the most vulnerable patients who are difficult to contact due to communication barriers.

In conclusion, adult CRS patients use a combination of methodology, preexisting rationale, and emotional response to guide their decision to seek unplanned postdischarge care. They primarily identify clear expectations for discharge, clear communication, ease of communication, ease of access to care, and reliable and ongoing support as targets for intervention to reduce unplanned postdischarge care and facilitate well-coordinated, lower cost care.

## Supplemental Material

sj-pdf-1-qhr-10.1177_10497323211002479 – Supplemental material for Understanding Patients’ Decisions to Obtain Unplanned, High-Resource Health Care After Colorectal Surgery Supplemental material, sj-pdf-1-qhr-10.1177_10497323211002479 for Understanding Patients’ Decisions to Obtain Unplanned, High-Resource Health Care After Colorectal Surgery by Stephanie T. Lumpkin, Eileen Harvey, Paul Mihas, Timothy Carey, Alessandro Fichera and Karyn Stitzenberg in Qualitative Health Research

sj-pdf-2-qhr-10.1177_10497323211002479 – Supplemental material for Understanding Patients’ Decisions to Obtain Unplanned, High-Resource Health Care After Colorectal Surgery Supplemental material, sj-pdf-2-qhr-10.1177_10497323211002479 for Understanding Patients’ Decisions to Obtain Unplanned, High-Resource Health Care After Colorectal Surgery by Stephanie T. Lumpkin, Eileen Harvey, Paul Mihas, Timothy Carey, Alessandro Fichera and Karyn Stitzenberg in Qualitative Health Research

## Notes

1 Interviewer A was a female postdoctoral (MD) research fellow and general surgery resident with qualitative interviewing training. Interviewer A completed the first 15 of the 18 interviews. Notably, Interviewer A did not have any overlapping clinical responsibilities or clinical relationship with the enrolled participants in this study. Still, as interviews progressed, we assessed for diversity of responses and noted concern for a social desirability bias regarding the patient–surgeon relationship. For this reason, a second, nonphysician interviewer (B) completed the remaining three interviews with a medical outsider’s lens, and paid specific attention to questions regarding the relationship between the patient and the surgical team. Interviewer B was a female qualitative researcher with a bachelors in science in global studies, with a background in public health.
