# Supplementary material for: Understanding Patients’ Decisions to Obtain Unplanned, High-Resource Health Care After Colorectal Surgery
Source: Qual Health Res. 2021 Apr 10;31(9):1582–95. doi: 10.1177/10497323211002479 (PMC8438771; doi:10.1177/10497323211002479)
Supplement: sj-pdf-1-qhr-10.1177_10497323211002479 – Supplemental material for Understanding Patients’ Decisions to Obtain Unplanned, High-Resource Health Care After Colorectal Surgery [file sj-pdf-1-qhr-10.1177_10497323211002479.pdf]

Sample Semi-Structured Interview Script

Introduction: Hello, Mr. or Ms. X, My name is [ ] and I am a researcher at [Institution] and I am contacting you regarding your recent surgery. I have your written consent, but participation is voluntary and you can choose to leave the study at any time. This interview will last 45-60 minutes, is now an ok time to talk? I would like to start with a few brief questions.

Pre-Screen – Ask all patients

1. Since your surgery, have you seen or communicated with your surgeon or surgical team?
2. Since your surgery, have you been hospitalized, readmitted, or visited an emergency room? Where?
3. How would you rate your postoperative course? Straight-forward – A few bumps in the road – Complex – Nightmare. Tell me more about that?

Patient Health Questionnaire 2-Item Depression Screen[1]

1. In the past 2 weeks, how frequently have you had a depressed mood?
    a. Not at all (0), several days (1), more than half the days (2), or nearly every day (3)
2. In the past 2 weeks, how frequently have you had a lack of pleasure in usual activities?
    a. Not at all (0), several days (1), more than half the days (2), or nearly every day (3)

Brief Health Literacy Screener[2]

1. How confident are you filling out medical forms by yourself?
    a. Not at all, a little, somewhat, quite a bit, and extremely (1→5)
2. How often do you have problems learning about your medical condition because of difficulty understanding written information?
    a. Always, often, sometimes, rarely, or never (1→5)
3. How often do you have someone like a family member, friend, hospital or clinic worker or caregiver, help you read hospital materials?
    a. Always, often, sometimes, rarely, or never (1→5)

Based on above answers, stratify – UHRHU or No UHRHU

START RECORDING

Now I would like to move on to our less formal conversation. There are no right or wrong answers.

1. Tell me about your surgery and hospital stay. Why did you have surgery?
2. How did you feel about your preparation for discharge while in the hospital?
    a. Can you give an example of when you did not clearly understand what to expect after surgery?
    b. How would you contact your surgeon if you had a problem on a Saturday evening at 5 pm? A Tuesday morning at 9 am?
3. What concerns have you had about your health since leaving the hospital?
4. Describe your relationship with your surgeon.
5. *Tell me more about your communication with your surgical team.
6. Describe the day that you went to clinic for your follow-up visit, if you went to one.
    a. How did you get to your appointment?
    b. How does a planned doctor's appointment fit into your daily routine?
7. *If yes to Pre-screen 2 – Tell me more about your readmission/ED Visit.
    a. What was going through your mind when you saw this provider?
    b. Where was this provider located?
    c. Why did you choose this provider? What did they treat you for? Were you sent home?
    d. Do you think this could have been prevented?
    e. How would you compare the care that you received at this appointment compared to the care you would receive from your surgeon?
8. If no to Pre-screen 2 – Did you ever consider going to the ED?
    a. What would have made you go to the ED?
9. Who lives at home with you? Tell me more about how your relationships at home affect your health.
10. Does anyone come to appointments with you? Tell me more about how they influence your healthcare.
11. Can you describe any impact that this surgery or your condition may have had on your emotional state?
12. Can you describe any impact that this surgery may have had on your psychological state?

13. How has your mood impacted your ability to communicate with your surgeon after surgery?
14. If you could have addressed your problem in clinic instead of the emergency room or readmission, would you chosen to have your problem addressed completely in clinic?
    a. What could have been done to prevent your ED visit or readmission?
    b. What prevented you from visiting your surgeon's clinic prior to your readmission or ED visit?
15. Is there anything that you wish could have been done differently since you left the hospital? Can you tell you more about that?

References

1. www.cqaimh.org/pdf/tool_phq2.pdf.

2. Sarkar U, Schillinger D, López A, Sudore R. Validation of self-reported health literacy questions among diverse English and Spanish-speaking populations. *J Gen Intern Med*. 2011;26(3):265-271. doi:10.1007/s11606-010-1552-1
