# Supplementary material for: Understanding Patients’ Decisions to Obtain Unplanned, High-Resource Health Care After Colorectal Surgery
Source: Qual Health Res. 2021 Apr 10;31(9):1582–95. doi: 10.1177/10497323211002479 (PMC8438771; doi:10.1177/10497323211002479)
Supplement: sj-pdf-2-qhr-10.1177_10497323211002479 – Supplemental material for Understanding Patients’ Decisions to Obtain Unplanned, High-Resource Health Care After Colorectal Surgery [file sj-pdf-2-qhr-10.1177_10497323211002479.pdf]

Supplemental Table: Characteristics of Persons According to Decision-Making Themes in Semi-Structured In-Depth Interviews (n=18)

Characteristic	Algorithmic (N=5)	Guided (N=9)	Impulsive (N=4)
	Median (IQR) or Percent		
Age in years	49 (49-69)	63 (62-65)	46 (39-50)
Female, %	60%	78%	0%
Race %			
White	80%	78%	100%
Non-White	20%	22%	0%
Driving distance in miles	32 (5-44)	63 (34- 81)	200 (101-510)
Primary Insurance, %			
Private	80%	67%	75%
Public or Self-Pay	20%	33%	25%
Indication for surgery, %			
Neoplasm	80%	22%	25%
Inflammatory Bowel Disease	0%	0%	75%
Diverticulitis	0%	11%	0%
Obstruction or Volvulus	0%	33%	0%
Other	20%	33%	0%
In-Hospital Complication, %	20%	22%	0%
ERAS Pathway, %	80%	78%	75%
Same Day Surgery, %	100%	67%	100%
Clinic appointment scheduled	80%	100%	100%
Length of Stay in days	5 (4-8)	5 (4-8)	4 (3-5)
Primary Procedure Type, %			
Colon Resection	100%	22%	75%
Rectal Resection	0%	22%	56%
Other	75%	0%	25%
Ostomy Created, %	20%	56%	50%
Surgical Approach, %			
Laparoscopic	20%	0%	25%
Robotic	60%	11%	50%
Open	20%	89%	25%
Depressive Symptoms Present [A]	0%	22%	75%
Inadequate Health Literacy [B]	20%	22%	0%
Non-routine postdischarge care [C]	40%	56%	25%
Unplanned event at non-index hospital	20%	22%	75%

Footnote Supplemental Table: Characteristics of interview respondents stratified by decision-making style – algorithmic, guided, or impulsive. [A] Depressive Symptoms were assessed using the Patient Health Questionnaire 2-Item Survey. [B] Health literacy was assessed based on the Brief Health Literacy Screen. [C] Non-routine postdischarge care included readmissions, emergency department visits, or observation stays at either the index hospital or a different hospital.
